# Suppression of Cone-Beam Artefacts with Direct Iterative Reconstruction Computed Tomography Trajectories (DIRECTT)

**DOI:** 10.3390/jimaging7080147

**Published:** 2021-08-15

**Authors:** Sotirios Magkos, Andreas Kupsch, Giovanni Bruno

**Affiliations:** 1 Bundesanstalt für Materialforschung und -prüfung (BAM), Unter den Eichen 87, 12205 Berlin, Germany; andreas.kupsch@bam.de (A.K.); giovanni.bruno@bam.de (G.B.); 2 Institute of Physics and Astronomy, University of Potsdam, Karl-Liebknecht-Str. 24-25, 14476 Potsdam, Germany

**Keywords:** iteration method, signal processing, X-ray imaging, computed tomography

## Abstract

The reconstruction of cone-beam computed tomography data using filtered back-projection algorithms unavoidably results in severe artefacts. We describe how the Direct Iterative Reconstruction of Computed Tomography Trajectories (DIRECTT) algorithm can be combined with a model of the artefacts for the reconstruction of such data. The implementation of DIRECTT results in reconstructed volumes of superior quality compared to the conventional algorithms.

## 1. Introduction

**Figure 1:**
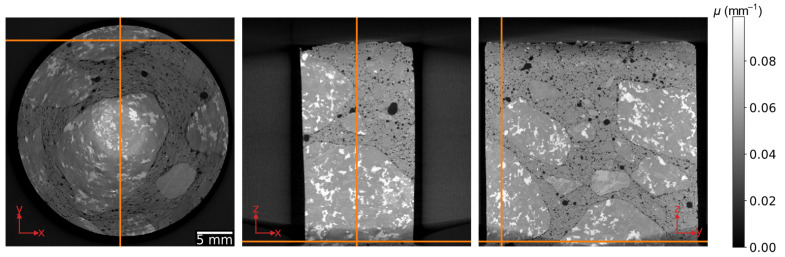
Orthogonal slices through the volume of a concrete rod as reconstructed by Feldkamp, Davis and Kress (FDK). The orange lines indicate the relative position of the cross sections.

Computed tomography (CT) systems for non-destructive testing and material analysis generally use a cone beam on a sample that rotates in a circular orbit [1], with cylindrical samples being among the most common [1,2,3,4]. The exact reconstruction of data acquired during such a measurement is not possible because the geometry does not satisfy Tuy’s sufficiency condition [5]. This is demonstrated in Figure 1 with the reconstruction of a concrete rod by the commonly used algorithm developed by Feldkamp, Davis and Kress (FDK) [6]. For higher cone angles, there is a decrease of the grey values, which represent the attenuation coefficient *μ*, and of the image quality in the direction of the rotation axis (*z*-axis in Figure 1).

Several algorithms have been proposed to reduce such artefacts. Hsieh proposed a two-pass algorithm that estimates the cone-beam artefacts from the segmented high-density material and then subtracts them from the FDK reconstruction [7]. Han and Baek went further by devising a multi-pass approach that they tested for larger cone angles and different material densities [8]. Maaß et al. proposed an iterative algorithm that also subtracts the estimated artefacts from the FDK reconstruction without requiring segmentation [9].

Here, we will describe how we have adjusted the Direct Iterative Reconstruction of Computed Tomography Trajectories (DIRECTT) algorithm [10,11,12] to estimate such artefacts and compensate for them.

## 2. Materials and Methods

### 2.1. Sample Images

A set of 3000 cone-beam projections of the concrete rod of Figure 1 was acquired over 360° on an in-house GE v|tome|x L 300 scanner. A 2024 × 2024 PerkinElmer detector with a pixel size of 0.2 mm was used. Source-object and source-detector distances of 81 mm and 1018 mm, respectively, resulted in a magnification of 12.5 for a voxel size of 0.016 mm. The voltage and current settings of the source were set to 140 kV and 80 μA, respectively. A 0.5 mm Cu prefilter was used. The acquisition time per projection was 6 s.

**Figure 2:**
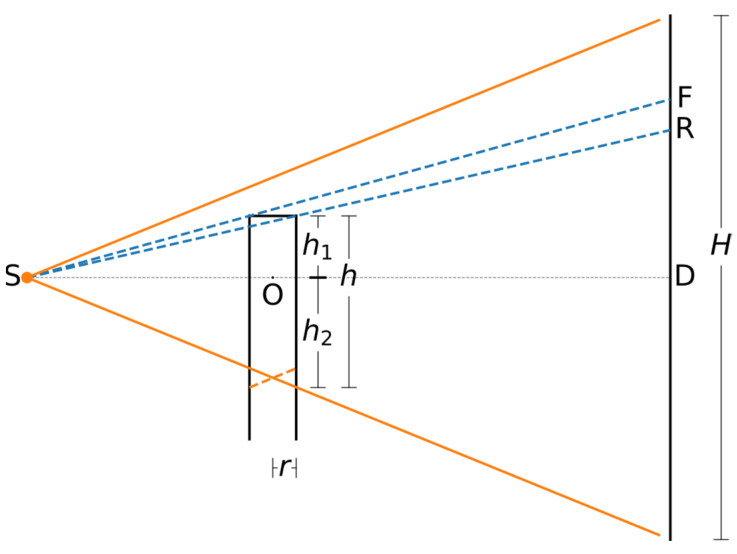
Geometric representation of the computed tomography (CT) scan (symmetric with respect to the central plane SOD).

The geometry of the CT scan of the cylindrical sample is represented, not to scale, by Figure 2. The orange cone represents the field of view (FoV), while the blue dashed lines represent rays that traverse the front and rear edges of the sample. Near the lower edge of the FoV, an inverse conical area of the sample is defined by the solid and dashed orange lines. This is the part of the sample that lies within the FoV during only some of the projections and, therefore, is not fully reconstructible by FDK [13].

We consider the case that dimensions of the sample are not known precisely. It is possible to determine them from the projections. The total height *h* of the sample within the FoV is the sum of its parts *h*_1_ and *h*_2_ that extend respectively above and below the plane SOD. The plane is defined by the source (S), the centre of rotation (O) and the centre of the detector (D). The plane SOD is assumed to be perpendicular to the detector.

We can see from Figure 2 that,

(1) $$\frac{\mathrm{SO}-r}{\mathrm{SD}}=\frac{h_{1}}{\mathrm{DF}}$$

and,

(2) $$\frac{\mathrm{SO}+r}{\mathrm{SD}}=\frac{h_{1}}{\mathrm{DR}}$$

where *r* is the radius of the sample, and DF and DR the distance between the central detector row and the detector rows where the front (F) and rear (R) edge of the concrete rod are respectively projected.

Dividing Equation (1) by Equation (2) and rearranging, we obtain:

(3) $$r=\frac{\mathrm{DF}/\mathrm{DR}-1}{\mathrm{DF}/\mathrm{DR}+1}\cdot \mathrm{SO}.$$

The length *h*_1_ can be calculated now from either Equation (1) or Equation (2), while the length *h*_2_ is:

(4) $$h_{2}=\frac{\mathrm{SO}+r}{\mathrm{SD}}\cdot \frac{H}{2}$$

where *H* is the height of the detector. The part of the sample that, as mentioned above, does not always lie within the FoV has been accounted for through the inclusion of the radius *r* in Equation (4).

### 2.2. The Direct Iterative Reconstruction of Computed Tomography Trajectories (DIRECTT) Algorithm

The DIRECTT algorithm was first proposed for the reconstruction of two-dimensional (2D) images by Lange et al. and, in a previous article [12], we introduced a new, more efficient, and fully 3D version. The algorithm operates on finding the best solution possible by mimicking the actual physical projection process, instead of directly solving the inverse problem. It only reconstructs certain voxels during each iteration, simulating the projection of the partial reconstruction, and repeating the workflow for the difference between measured and simulated projections until this difference is sufficiently close to zero [10,11,12]. Although the concrete rod of Figure 1 was one of the two datasets that were used to showcase the performance of DIRECTT, the algorithm was implemented only on the slice that corresponds to the cross section of the sample with the plane SOD [12]. That slice will be hereafter referred to as the central slice. Attempting to implement DIRECTT on the whole dataset does not lead to an improvement over the FDK reconstruction of Figure 1. On the contrary, it results in severe artefacts and missing data because the algorithm fails to predict the decreasing grey values along the *z*-axis. However, these artefacts can be reduced by modelling them based on the shape of the sample.

### 2.3. Software

For this work, the forward- and back-projection operations involved in DIRECTT were performed using the Python programming language and the open-source ASTRA (All Scale Tomographic Reconstruction Antwerp) toolbox [14]. Via ASTRA, computationally demanding operations are offloaded to a graphics processing unit using the CUDA (Compute Unified Device Architecture) language. The toolbox also includes several reconstruction algorithms, such as the FDK, the simultaneous iterative reconstruction technique (SIRT) [15], and a conjugate gradient (CG) method based on the Krylov subspace [16], that can run with little input from the user [14].

## 3. Results

**Figure 3:**
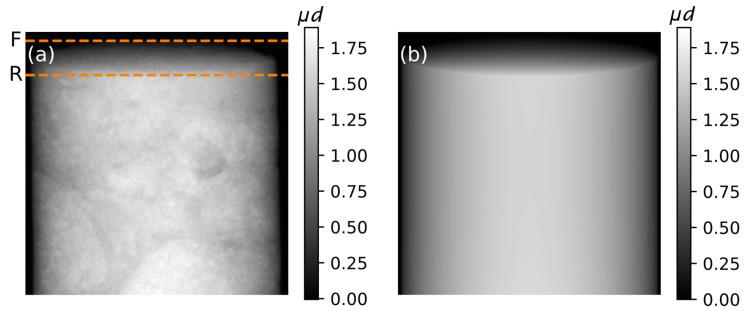
(**a**) Projection of the concrete rod. The detector rows on which the front (F) and rear (R) edge of the concrete rod are respectively projected are marked by horizontal lines. (**b**) Simulated projection of a homogeneous cylinder of dimensions equal to the rod. The greyscale values in both figures correspond to the attenuation integral values *μd*.

A single measured projection of the concrete and the corresponding simulated projection of a virtual homogeneous cylinder of height *h* and radius *r* are shown in Figure 3a,b, respectively. The two horizontal lines in the former indicate the detector rows that correspond to points F and R of Figure 2. The two rows are identified automatically from the projections. Specifically, F is the lowermost detector row containing exclusively values that correspond to the background. Similarly, R is the lowermost row containing exclusively values lower than the mode of all absorption projections, which roughly corresponds to the absorption of rays that penetrate the sample perpendicularly.

**Figure 4:**
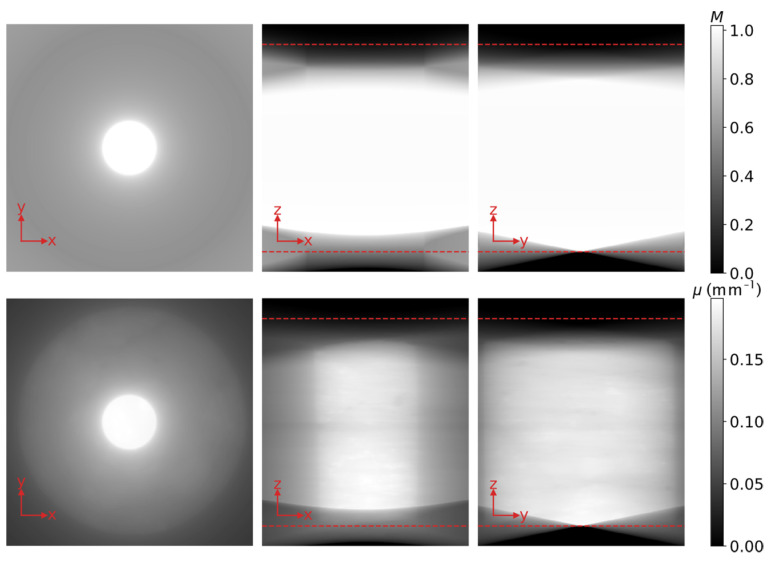
Top row: Orthogonal slices through the model *M* of the artefacts that arise during the reconstruction; Bottom row: Orthogonal slices through the volume resulting from the back-projection of the unfiltered projections of the concrete rod. The extent of the volume of Figure 1 along the z-axis is indicated by the red dashed lines. Apart from that, the position of the slices shown in either row corresponds precisely to that of the slices in Figure 1.

By simulating the geometry of the scan, projecting and back-projecting the virtual cylinder, and normalizing for the central slice, a 3D model *M* of the artefacts that arise during the reconstruction is computed. Normalizing for the central slice ensures that hardly any artefacts are considered for the parts of the volume that always lie within the FoV and can be accurately reconstructed by FDK. Note that the model *M* needs to be computed just once. The slices of *M* that correspond to those in Figure 1 are shown in the top row of Figure 4. The volume resulting from the back-projection of the unfiltered projections of the concrete rod, the first step during each iteration of DIRECTT [12], is shown in the bottom row of Figure 4. Both volumes of Figure 4 comprise more slices along the *z*-axis than that of Figure 1. The extra slices have been included because it is essential for the implementation of any iterative reconstruction algorithm, such as DIRECTT, that the parts of the sample that do not lie within the FoV for every projection are reconstructed too.

**Figure 5:**
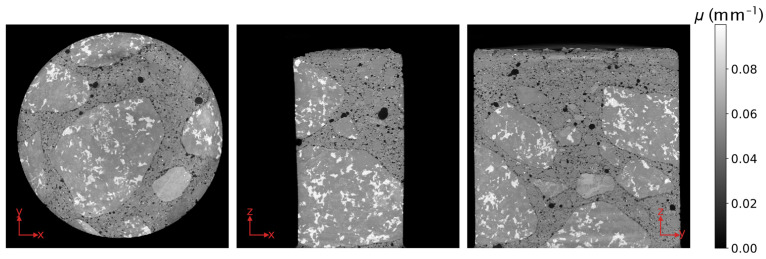
Final reconstruction of the concrete rod as computed by DIRECTT. The volume has the same size as the volume shown in Figure 1 through omission of its upper and lower slices. The position of the slices shown corresponds precisely to that of the slices in Figure 1.

There is an obvious qualitative relation between the two rows of Figure 4. The pixel-by-pixel division of the reconstructed volume by the model *M* results in a “corrected” volume, on which DIRECTT can be successfully implemented. The threshold values, based on which the voxels to be reconstructed during each iteration are selected, are calculated from the central slice as described in [12] but are applied simultaneously on the whole volume. The algorithm terminates when the projections of the reconstruction array match the measured ones. The final reconstructed volume is shown in Figure 5.

## 4. Discussion and Conclusions

The reconstruction by DIRECTT is an improvement over that by FDK in Figure 1 as there are no artefacts linked to the increasing cone angle. Lacking the ground truth for the reconstructed volume, the evaluation of the two algorithms using a full-reference metric is meaningless. Nevertheless, a quantitative evaluation of the algorithms can be undertaken by calculating the histogram entropy of the respective volumes. The histogram entropy (HE) is a global metric defined according to the relation:

(5) HE=∫p(μ)log[p(μ)]dμ,

where *p*(*μ*) is the distribution function of the grey values. The value of the HE increases if homogeneously distributed noise is present in the image, and decreases if sharp edges are present [17]. Therefore, a low value is an indication of a good balance between noise and blur. The FDK- and DIRECTT-reconstructed volumes (Figure 1 and Figure 5, respectively) have histogram entropies of 2.86 and 1.89, respectively.

**Figure 6:**
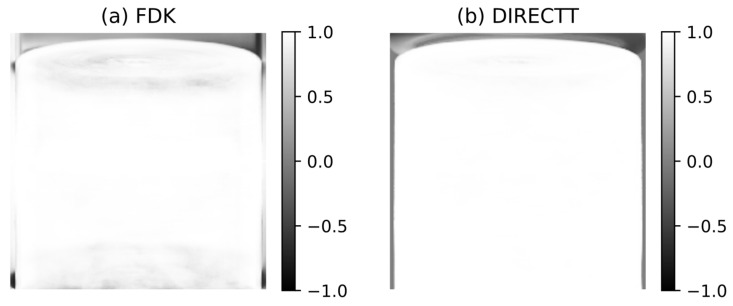
Pearson correlation coefficient between measured projections and simulated projections of the volumes reconstructed by: (**a**) FDK, and (**b**) Direct Iterative Reconstruction of Computed Tomography Trajectories (DIRECTT). The Pearson correlation coefficient (PCC) has been calculated for each detector pixel.

An alternative way to evaluate the results of the algorithms is to simulate the projection of the reconstructed volumes and calculate the Pearson correlation coefficient [12,18] between these projections and the measured ones. The Pearson correlation coefficient (PCC), the value of which can range between 1 for total linear correlation and −1 for total linear anti-correlation, is calculated according to the relation:

(6) $${\mathrm{PCC}}_{M,P}=\frac{\sigma_{M,P}}{\sigma_{M}\sigma_{P}},$$

where σ_M,P_ is the covariance and σ_M_ and σ_P_ are the standard deviation of the measured and simulated projections, respectively. Such coefficients are calculated for each detector pixel and are plotted in Figure 6. While the PCC values decrease for large cone angles in the case of FDK, they remain near 1 regardless of the angle in the case of DIRECTT.

**Figure 7:**
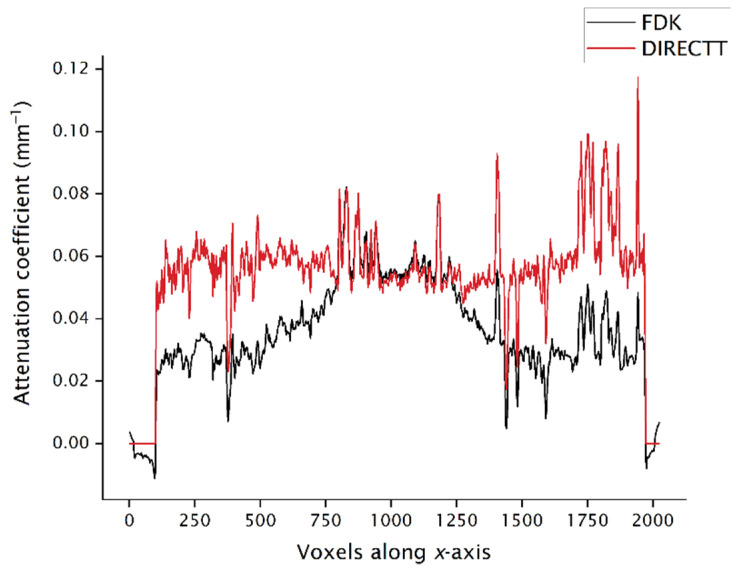
Profile along the *x*-axis of the attenuation coefficient values of the first slice shown in both Figure 1 and Figure 5.

The comparison between the two algorithms can also be done locally on parts of the volume that are affected in a greater degree by the cone-beam artefacts. For instance, the profiles along the *x*-axis through the centres of the *xy*-slices in both Figure 1 and Figure 5 are plotted in Figure 7. It is evident that, in the case of FDK, the attenuation values away from the centre of the sample are underestimated.

**Figure 8:**
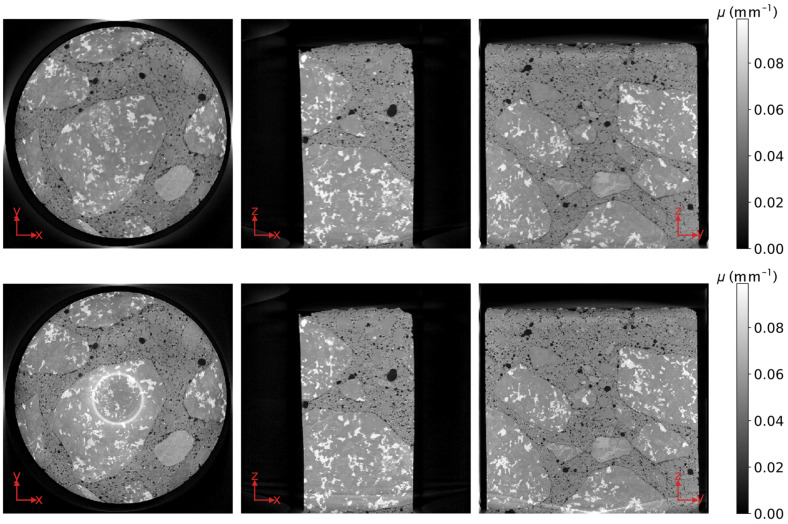
Orthogonal slices through the volume of a concrete rod as reconstructed by simultaneous iterative reconstruction technique (SIRT, top row) and conjugate gradient (CG, bottom row). The volume has the same size as the volume shown in Figure 1 through omission of its upper and lower slices. The position of the slices shown corresponds precisely to that of the slices in Figure 1.

Finally, the reconstruction of the volume has also been performed using SIRT and CG, which were programmed to perform a fixed number of 600 iterations. The results are shown in Figure 8. The values of the metrics for each reconstruction algorithm are listed in Table 1. While the simulated projections in the case of both SIRT and CG have a high correlation to the measured ones, the respective histogram entropy values are significantly higher than that of DIRECTT. Moreover, although CG has, for the better part, suppressed the artefacts that are linked to the increasing cone angle, it has, at the same time, resulted in ring-like artefacts exactly where the regions that lie fully within the FoV border the regions that are only partially within it.

To sum up, we have described how the DIRECTT algorithm is adjusted for the reconstruction of cone-beam CT data. The artefacts that normally arise during such an operation have been suppressed resulting in a reconstruction that is a clear improvement over that computed by FDK. The performance of DIRECTT has been quantified by both global and local metrics and compared to the performance of other iterative reconstruction algorithms.

## Figures and Tables

**Table 1:** Comparison of the performance of the reconstruction algorithms.

Reconstruction Algorithm	Number of Iterations	Average Time per Iteration (s) ^1^	Histogram Entropy	Mean Value of PCC
FDK	1	54.85 ± 0.26	2.86	0.75
DIRECTT	562	14.51 ± 0.18	1.89	0.92
SIRT	600	6.15 ± 0.05	2.61	0.97
CG	600	6.41 ± 0.01	2.33	0.97

^1^ On a computer equipped with an NVIDIA GeForce GTX 1080 Ti GPU.

## Data Availability

Restrictions apply to the availability of these data. Data were obtained from BAM and are available from the authors with the permission of BAM.
